# Belt buckle ectropion: a distinct mechanism of lower eyelid malposition after Mohs reconstruction

**DOI:** 10.3389/fopht.2026.1804372

**Published:** 2026-03-25

**Authors:** Gokce Cinel Pasa, Addison M. Demer, Lilly H. Wagner

**Affiliations:** 1 Department of Ophthalmology, Mayo Clinic, Rochester, MN, United States; 2 Department of Dermatology, Division of Dermatologic Surgery, Mayo Clinic, Rochester, MN, United States

**Keywords:** complication, ectropion, eyelid, Mohs surgery, oculoplastic surgery, reconstruction

## Abstract

Belt buckle ectropion is a newly recognized mechanism of lower eyelid malposition following Mohs reconstruction. Unlike cicatricial ectropion caused by vertical skin shortage, this variant results from horizontal flap tension acting on a lax tarsus. We retrospectively reviewed three patients who developed postoperative ectropion following subciliary Mohs reconstruction without lid margin involvement. Mechanism, management, and outcomes were analyzed. In all cases, the ectropion resulted from horizontal anterior lamellar tension producing anterior tarsal tilt, confirmed by a negative forced traction test. One case resolved spontaneously, and two required lateral tarsal strip procedures, both yielding successful anatomic and functional outcomes. Belt buckle ectropion represents a distinct post-reconstructive malposition mechanism, correctable with horizontal tarsal tightening rather than skin grafting. Recognition of this pattern may prevent misclassification as cicatricial ectropion and guide appropriate surgical management.

## 1 Introduction

This report defines belt buckle ectropion as a newly recognized form of post-reconstructive lower eyelid malposition resulting from horizontal tension of the anterior lamella in patients with pre-existing tarsal laxity. Unlike classic cicatricial ectropion, which is caused by vertical skin shortage and typically requires correction with skin graft, belt buckle ectropion is caused by forward rotation of the tarsus due to a band of horizontal tension along its inferior border, without vertical deficiency, and can be corrected with horizontal tarsal tightening.

The recognition of this distinct mechanism is important to differentiate the mechanical causes of ectropion from cicatricial ones and guide appropriate surgical management.

## 2 Methods

We describe three patients who developed ectropion following reconstruction of partial-thickness Mohs defects of the subciliary eyelid skin, without lid margin involvement. Reconstruction was performed by the same oculoplastic surgeon in two cases and by a Mohs surgeon in one case with horizontally oriented skin advancement flaps. One case improved spontaneously, while two did not resolve over a 3-month observation period and required correction. Neither patient had vertical skin shortage, as evidenced by the ability to advance the lower lid over the cornea with digital traction (“forced traction test”). The mechanism of ectropion was determined to be horizontal flap tension producing anterior tarsal tilt—a mechanical action akin to the pull of a belt buckle under a protruding abdomen.

This study was conducted in accordance with the ethical standards of the 1975 Declaration of Helsinki. Institutional review board approval was not required for this retrospective case series. Written informed consent for the publication of clinical photographs was obtained from all subjects included in the report.

## 3 Results

### 3.1 Case 1

An 88-year-old woman underwent Mohs excision of a nodular basal cell carcinoma involving the junction of the left nasal sidewall and lower eyelid. The final defect was reconstructed using a bilobed glabellar flap combined with a horizontal advancement flap based at the lateral lower lid. Rotation of the lid margin was noted during surgery but was expected to improve as tension on the flaps eases postoperatively.

**Figure 1:**
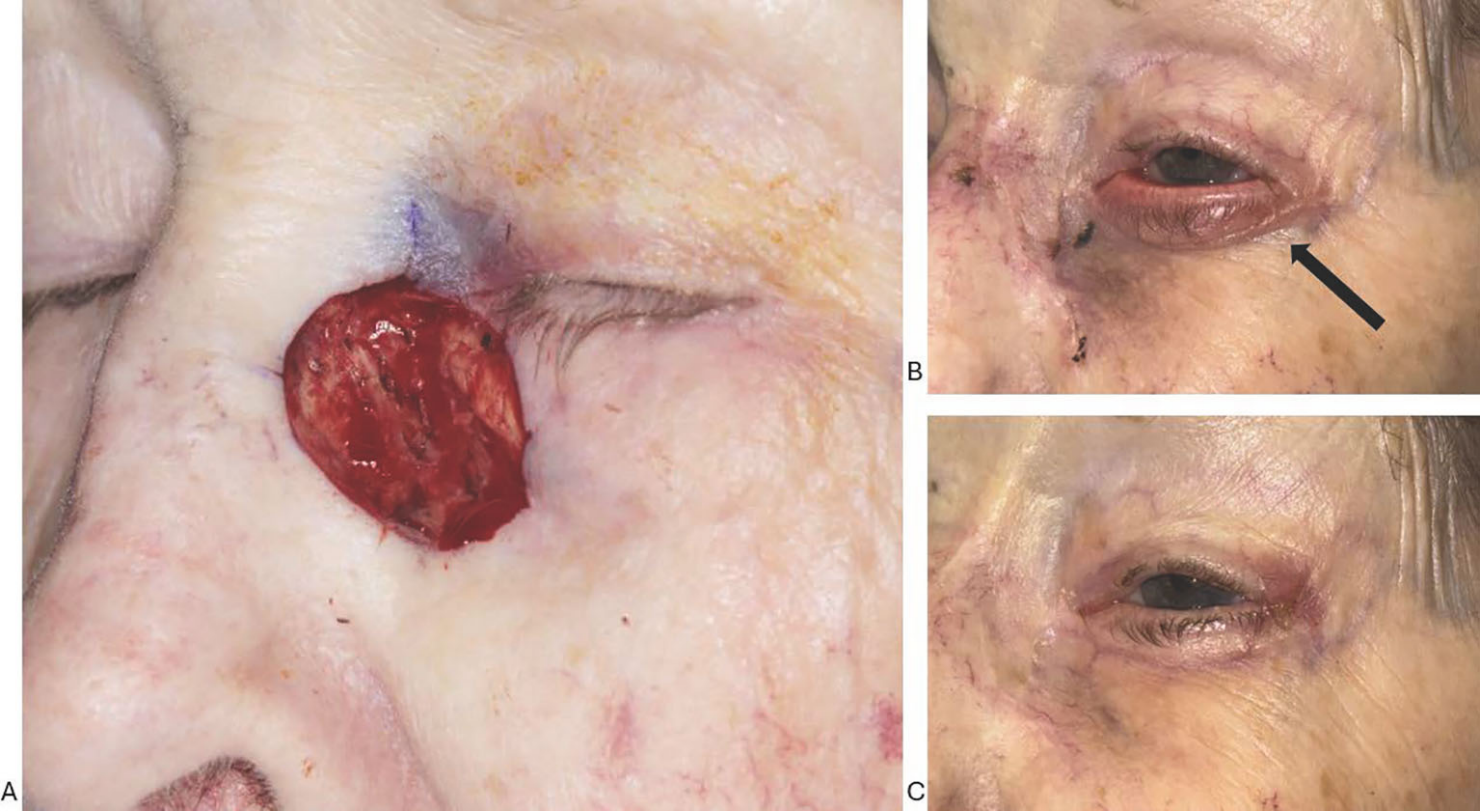
**(A–C)** Patient 1 was referred for the closure of a 2.6 × 2.2-cm defect involving the nasal sidewall, medial canthus, and nasal lower lid **(A)**. Early after repair with bilobed glabellar flap and horizontal lower lid advancement flap, she developed left lower eyelid ectropion with anterior tarsal tilt and punctal eversion **(B)**, which did not resolve over several months of observation. Note the horizontal line of tension along the inferior tarsal border (black arrow). Following a lateral tarsal strip procedure, the lower eyelid position is fully corrected **(C)**.

At 2 weeks postoperatively, she was noted to have lower lid ectropion, but no evidence of vertical skin deficit or excessive scar contraction. An examination revealed anterior tilt of the tarsus due to horizontal skin flap tension—a presentation consistent with belt buckle ectropion (**Figures 1A, B**). After conservative management with ocular lubrication and observation for 3 months, the eyelid malposition persisted, and the patient was bothered by chronic eye discomfort and watering.

At 4 months post-reconstruction, the patient underwent a lateral tarsal strip procedure. Horizontal tightening of the lower eyelid at the level of the tarsus corrected the anterior tilt without need for a skin graft. The postoperative outcome was successful both anatomically and functionally, with restoration of normal eyelid position and resolution of ocular exposure symptoms (**Figure 1C**). The lid position remained excellent at final follow-up 6 months after the ectropion repair.

### 3.2 Case 2

**Figure 2:**
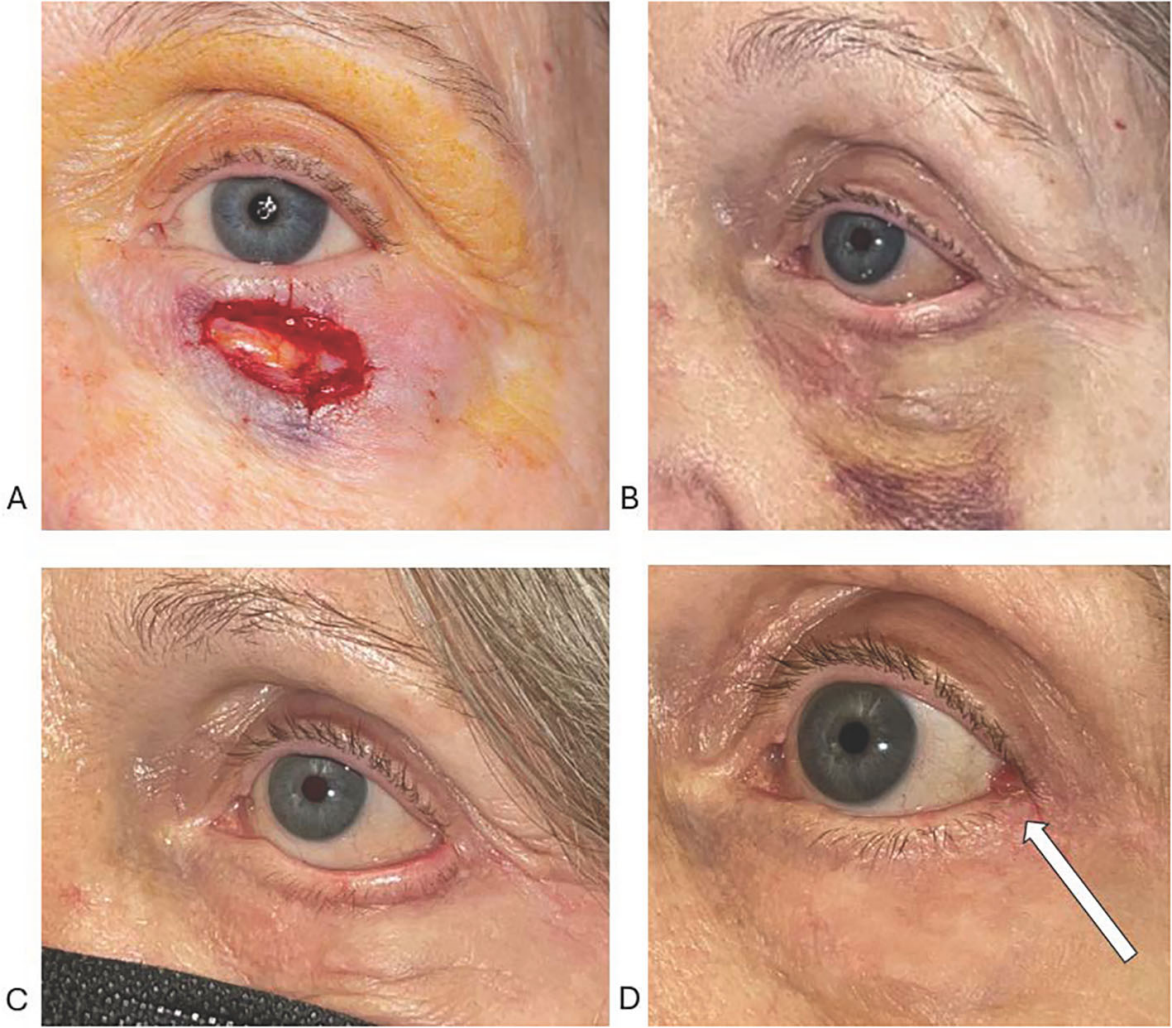
**(A–D)** Non-margin involving Mohs defect **(A)** that was repaired with horizontal O-to-Z flaps. Lower lid retraction and ectropion at the initial post-operative visit 2 weeks after reconstruction **(B)**, which persisted for 2 months **(C)**. The lower lid could easily be advanced over the cornea with manual upward traction, proving the absence of vertical skin shortage. Revision with lateral tarsal strip restored the normal lower lid contour **(D)**. Note the small pyogenic granuloma at the lateral commissure (white arrow); this was excised in the office.

An 80-year-old woman underwent Mohs excision of a basal cell carcinoma involving the central and lateral aspect of the left lower eyelid. The resulting anterior lamella defect (**Figure 2A**) was reconstructed with horizontal O-to-Z advancement flaps. Lower lid ectropion and retraction were noted at her 2-week postoperative visit. The malposition was attributed to flap-induced horizontal tension acting on a structurally lax tarsus. There was no vertical skin deficit or vertically oriented tight scar bands (**Figure 2B**).

After an additional 10 weeks of observation without improvement of the lid position and ocular exposure symptoms, the patient elected to have correction with a lateral tarsal strip procedure. Tightening of the lax tarsus alone restored the lid-globe apposition. At 3-months follow-up after ectropion correction, the patient’s eyelid position remained stable (**Figures 2C, D**). A small postoperative pyogenic granuloma at the lateral canthus was excised in the office.

### 3.3 Case 3

**Figure 3:**
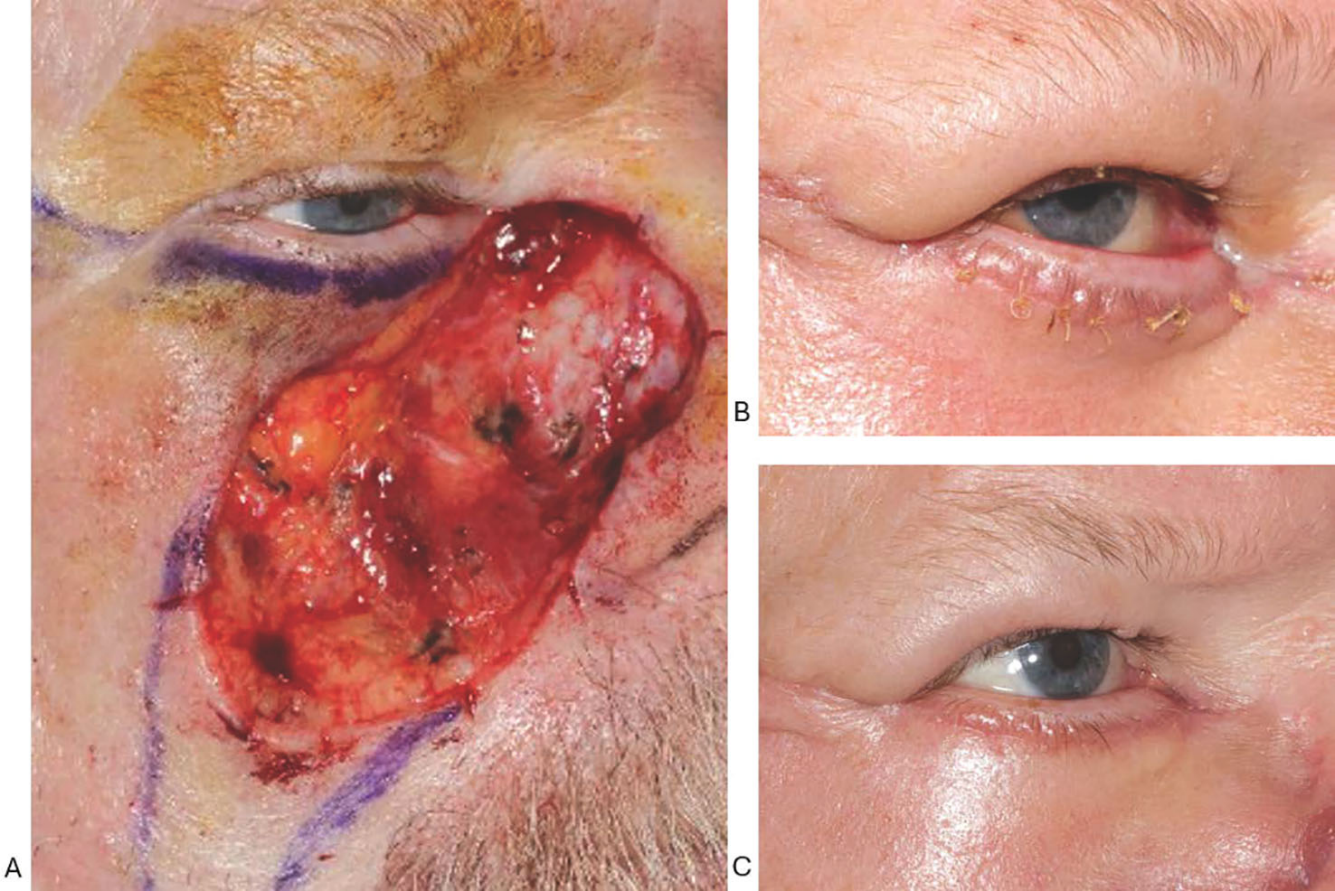
**(A–C)** Large midface defect involving the cheek, subciliary lower lid, and nasal sidewall **(A)** that was repaired with temporally based advancement flap. Belt buckle ectropion was noted intraoperatively, and the position of periosteal tacking sutures was adjusted to mitigate excessive pre-tarsal flap tension and redirect the vector more diagonally. The ectropion that was observed at postoperative week 1 **(B)** had spontaneously improved by postoperative month 1 **(C)**.

A 61-year-old male patient had Mohs surgery for a 3.5 × 3-cm nodular basal cell carcinoma of the upper cheek. The final defect involved the nasal lower eyelid up to the lash line (**Figure 3A**). Reconstruction was done with a large temporally based rotation flap, and belt buckle ectropion was noted intraoperatively. To mitigate excessive horizontal flap tension along the inferior border of the tarsus, the flap was secured to the periosteum of the zygoma at the superior aspect of the lateral orbital, redirecting the tension more diagonally, and tension on the temporal part of the flap, closer to the base, was maximized with periosteal fixation sutures in order to decrease tension on the pre-tarsal part.

Although there was ectropion with belt buckle mechanism at the postoperative week 1 visit, this had spontaneously improved by postoperative month 1 (**Figures 3B, C**). No further corrective surgery was needed.

## 4 Discussion

The “belt buckle” ectropion mechanism is distinguishable by (1) anterior lamella-only defect (2), horizontal vector flap tension (3), pre-existing tarsal/lower eyelid laxity, and (4) absence of vertical skin shortage or scar bands, and it responds to horizontal tarsal shortening procedures.

Preoperative evaluation of eyelid laxity and careful flap planning are essential to avoid eyelid malposition after Mohs reconstruction. Horizontal eyelid tone should be assessed in all patients prior to periocular skin cancer surgery with snap-back test, and eyelid tightening should be considered if snap back is moderately decreased to poor (i.e., lower lid does not return to normal position without blinking when manually distracted from the eye). The risks of ectropion and lid retraction due to excessive vertical traction are well described in the lower eyelid, especially in patients with pre-existing lower lid laxity, lid–cheek junction defects, or a negative orbital vector (1–5). These studies emphasize the different mechanisms of ectropion development and potential multifactorial nature and highlight the relevance of distinguishing cicatricial causes from mechanical ones. The importance of orienting skin tension parallel to the lid margins, which may allow direct closure of even large defects, is a well-known principle in eyelid reconstruction (6).

Our case series demonstrates the relevance of vector forces in post-reconstructive lower eyelid malposition and introduces “belt buckle ectropion” as a distinct entity. Unlike cicatricial ectropion—which arises from vertical skin shortage or scar contracture and requires skin grafting—belt buckle ectropion results from horizontal skin flap tension, causing tarsal tilt in the setting of pre-existing laxity, and can be corrected with tarsal tightening alone. Notably, all three patients had reconstruction with local flaps. The authors’ choice of reconstructive technique depends on the defect size and shape, local skin availability and quality, patient’s ability to return for follow-up at 5–7 days for the removal of the bolster over the skin graft, and suitable donor site. In the three cases described in this report, the defects were too large for direct closure without creating a large dog ear deformity with need for additional skin excision, and local flaps were felt to be the best option. However, alternative techniques should be considered when local flap tension cannot be mitigated.

Both patients who had corrective surgery for ectropion were experiencing significant exposure symptoms and epiphora. When horizontal tightening is needed, surgical technique has to be customized to individual eyelid anatomy. Overall tarsal laxity such as in floppy eyelid syndrome may require wedge excision, taking care to only excise the tarsus and not add to the anterior lamella defect, while lateral canthal tendon disinsertion with rounded and medially displaced lateral canthus is best corrected with lateral tarsal strip procedure. Intraoperative maneuvers may reduce the risk for persistent belt buckle ectropion, as demonstrated in case 3. These include redirection of the vector of maximum flap pull or redistribution of flap tension away from the pre-tarsal area.

Recognition of this mechanism is critical because mislabeling as cicatricial ectropion may lead to unnecessary skin grafting. In some cases, belt buckle ectropion may resolve spontaneously as flap tension decreases; in others, horizontal tightening (lateral tarsal strip or wedge excision) is required. Our series supports a tailored surgical approach to post-reconstructive ectropion based on diligent analysis of underlying mechanical forces. Larger series with 10 or more cases are needed for a more systematic analysis of best timing and technique for intervention.

## Data Availability

The raw data supporting the conclusions of this article will be made available by the authors, without undue reservation.

## References

[1] Moe KS Chang J Chundury RV Wong BJ . Ectropion following reconstruction of Mohs defects in the infraorbital cheek: incidence and risk factors. Arch Facial Plast Surg. (2013) 15:93–7. doi: 10.1097/MOO.0000000000000375, PMID: 28509671

[2] Silva AF Lima RS Macedo JLS . Analysis of risk factors for lower eyelid ectropion after Mohs micrographic surgery. Bras Dermatol. (2012) 87:899–903. doi: 10.1097/DSS.0000000000000125, PMID: 25229782

[3] Verity SM White CP White JB . Ectropion following reconstruction of periocular defects: the significance of lower eyelid laxity. Orbit. (2013) 32:287–91. doi: 10.1055/s-0040-1709515, PMID: 32413924

[4] Rames JD Rames MM Yu CY Sanchez Figueroa N Akpala CO Hussein S . Risk factors for ectropion after lower eyelid and cheek reconstruction following mohs micrographic surgery. Plast Reconstr Surg Glob Open. (2025) 13:e6498. doi: 10.1097/GOX.0000000000006498, PMID: 39906344 PMC11789856

[5] Sirek A Rootman DB Levin ML . Cicatricial lower eyelid ectropion: classification system and treatment algorithm. Ophthal Plast Reconstr Surg. (2015) 31:485–8. doi: 10.1055/s-0040-1709515, PMID: 32413924

[6] Thaller VT Vahdani K . The magic suture in periocular reconstruction. Eye (Lond). (2021) 35:2892–4. doi: 10.1038/s41433-020-01194-2, PMID: 32963313 PMC8452743

